# Periapical Lesion Grade and Sinus Mucosal Thickness in Severe Periodontitis

**DOI:** 10.1016/j.identj.2026.109683

**Published:** 2026-06-18

**Authors:** Wenyu Tang, Fanghui Chen, Pingchuan Qin, Xinrui Cui, Botakoz Xehesbek, Yahong Wen, Jizhou Wang, Ang Li, Chunhui Zhu

**Affiliations:** a Key Laboratory of Shaanxi Province for Craniofacial Precision Medicine Research, College of Stomatology, Xi’an Jiaotong University, Xi'an, Shaanxi, China; b Clinical Research Center of Shaanxi Province for Dental and Maxillofacial Diseases, College of Stomatology, Xi’an Jiaotong University, Xi'an, Shaanxi, China; c Department of Periodontology, College of Stomatology, Xi’an Jiaotong University, Xi’an, Shaanxi, China; d Department of Medical Imaging, College of Stomatology, Xi'an Jiaotong University, Xi'an, Shaanxi, China

**Keywords:** Severe periodontitis, Schneiderian membrane, Apical periodontitis, Cone-beam computed tomography

## Abstract

**Introduction and aims:**

Severe periodontitis (Stage III-IV) frequently coexists with apical periodontitis, both of which can contribute to Schneiderian membrane thickening (SMT). This study quantifies the association between periapical lesion severity and SMT in a well-defined cohort of severe periodontitis, aiming to improve the preoperative risk assessment using cone-beam computed tomography (CBCT).

**Methods:**

CBCT images from 330 patients diagnosed with Stage III-IV periodontitis complicated by apical periodontitis in maxillary posterior teeth were analysed. SMT was measured at standardised sites, and periapical lesion severity was graded using the CBCT periapical index (CBCT-PAI). Differences in SMT according to sex, periodontal stage and tooth position were evaluated, and correlations between CBCT-PAI and SMT were assessed using nonparametric tests and Spearman’s rank correlation.

**Results:**

Increased SMT was observed in both stage III and stage IV periodontitis, no significant difference between stages. SMT also exhibited a bilateral distribution pattern. Analysis of individual teeth revealed that the SMT showed a modest but statistically significant correlation with the severity of apical periodontitis (Stage III: ρ = 0.213, *P* < .001; Stage IV: ρ = 0.244, *P* < .001). Notably, SMT was particularly pronounced in the maxillary first molar (*P* < .001), which may reflect their close anatomical relationship with the sinus floor.

**Conclusion:**

In patients with severe periodontitis, SMT showed a modest association with the severity of coexisting apical periodontitis. Differences in SMT distribution were observed across different tooth positions; this may also be related to anatomical and functional factors.

**Clinical relevance:**

These findings provide supportive information for preoperative periodontal and dental assessments in patients requiring implant surgery, and may assist in preoperative risk assessment.

## Introduction

Periodontitis is a chronic biofilm-induced inflammatory disease characterised by progressive destruction of the periodontal supporting tissues.^1^ In advanced stages (Stage III-IV), substantial vertical bone loss frequently occurs in the posterior maxilla, often resulting in tooth loss and insufficient residual bone height for implant placement.^2^^,^^3^ In such cases, maxillary sinus floor elevation is commonly required prior to implant placement.^4^ The Schneiderian membrane (SM), which firmly adheres to the periosteum, has an average thickness of approximately 1 mm.^5^ A thickness exceeding 2 mm is generally considered pathological and has been associated with reduced elasticity, greater vascular fragility and a higher risk of membrane perforation.^6^^,^^7^ The perforation of SM is the most common intraoperative complication during sinus floor elevation procedure, with a reported incidence of approximately 20%, often resulting in surgical failure and implant instability.^8^

The roots of maxillary posterior teeth are in close proximity to the sinus floor, separated only by a thin cortical plate with a rich vascular and lymphatic supply.^9^ This anatomical proximity may facilitate the spread of odontogenic inflammation to the sinus mucosa, which may be associated with reactive thickening detectable on cone-beam computed tomography (CBCT).^9^ Odontogenic inflammation is a primary contributor to Schneiderian membrane thickening (SMT), which mainly arising from periodontal disease and apical periodontitis.^10^ Previous CBCT-based studies have demonstrated that the severity of periodontal conditions in maxillary molars influences the degree of SMT.^11^ Additionally, several studies have reported an association between periapical lesions and SMT, with approximately 80% of teeth affected by apical periodontitis exhibiting corresponding SMT, particularly in the molar region.^12^, ^13^, ^14^, ^15^ These conditions frequently coexist in patients with severely compromised dentitions, resulting in a complex inflammatory microenvironment in the posterior maxilla.^12^

Despite these findings, several notable gaps remain unaddressed. Most existing CBCT-based studies have evaluated periodontal disease or apical periodontitis separately, without considering their frequent coexistence in patients with advanced periodontal destruction. Furthermore, SMT has not been systematically investigated within a clearly defined periodontal staging framework, particularly in Stage III**–**IV periodontitis. Given that CBCT allows high-resolution visualisation of the tooth-bone–sinus complex, a standardised assessment in this high-risk cohort is clinically warranted.^13^

Therefore, this study aimed to address these gaps by focusing on a well-defined cohort of patients classified according to the 2018 World Workshop classification and by utilising the CBCT Periapical Index (CBCT-PAI) to evaluate the severity of periapical lesions. In addition, it investigates the association between severe periodontitis complicated by apical periodontitis and SMT. This research provides a preoperative assessment reference for maxillary sinus floor elevation in patients with periodontitis-related tooth loss, which may enhancing surgical success rates and postoperative stability.

## Material and methods

### Study population

This retrospective cross-sectional study was conducted in accordance with the ethical standards of the Declaration of Helsinki and was approved by the Ethics Committee of the Hospital of Stomatology, Xi’an Jiaotong University (Approval Number: KY-QT-20240001). All participants provided written informed consent before enrolment, and all data were anonymised prior to analysis.

CBCT scans and clinical records of patients who attended the Department of Stomatology, Xi’an Jiaotong University, between January 2024 and January 2026 were reviewed. From an initial cohort of 1050 patients who underwent CBCT for periodontal assessment, those meeting the following inclusion criteria were selected: (1) Stage III or Stage IV periodontitis according to the 2018 World Workshop classification; (2) age ≥18 years; (3) availability of diagnostic-quality CBCT covering bilateral maxillary sinuses and maxillary posterior dentition (second premolar-second molar); (4) presence of at least 1 posterior maxillary tooth with radiographic periapical changes. Exclusion criteria were as follows: (1) self-reported chronic sinonasal disease or systemic conditions likely to affect sinonasal mucosa (eg, COPD or asthma); (2) acute upper respiratory tract infection within 3 months; (3) history of previous sinus or maxillary surgery, maxillary posterior teeth with root fractures or extensive restorations precluding reliable CBCT assessment; (4) periodontal treatment within 6 months; (5) smoker. A total of 330 patients met the inclusion criteria and were included in the final analysis.

### CBCT acquisition protocol

All CBCT images were obtained using a HiRes3D cone-beam computed tomography system (HiRes3D). Patients were positioned with the Frankfort horizontal plane parallel to the floor. Imaging parameters were standardised at 100 kV, 4 mA, 15 s exposure time and a voxel size of 0.1 mm. The field of view was 16 × 8 cm. Images were reconstructed using medical imaging software (Mimics Medical, version 24.0; Materialize) for multiplanar reconstruction and analysis.

### Measurement procedures

SMT: The thickness of the SM was measured at 3 distinct locations per tooth – mesial, medial and distal – specifically for the maxillary second premolars, first molars and second molars. The maximum value among the 3 measurements was recorded as the SMT for each tooth position.^16^

The extent of periapical lesions: According to the new criteria established by Estrela et al, lesion diameters were measured in the axial, coronal, and sagittal planes and labelled as “a”, “b”, and “c”, respectively (Figure). The greatest diameter (*d*) was utilised to assign the CBCT-PAI grade as defined below: Grade 0 indicates a lesion diameter less than 0.5 mm; Grade 1 corresponds to a lesion diameter (*d*) greater than 0.5 mm and up to 1 mm; Grade 2 corresponds to *d* greater than 1 mm and up to 2 mm; Grade 3 corresponds to *d* greater than 2 mm and up to 4 mm; Grade 4 corresponds to *d* greater than 4 mm and up to 8 mm; and Grade 5 corresponds to *d* greater than 8 mm.^17^^,^^18^

**Fig.**
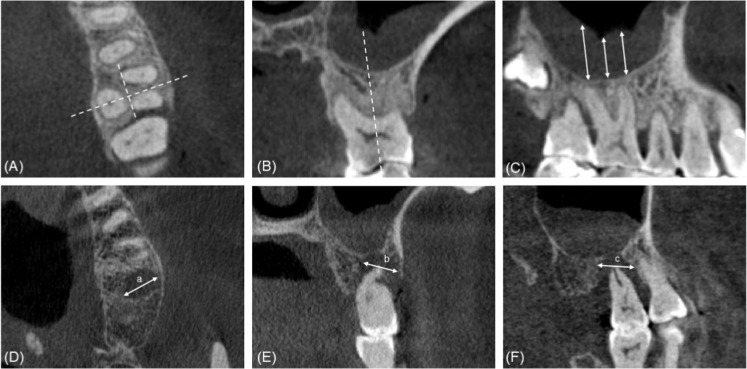
Image reconstruction and measurement protocol. (A) Axial view; (B) Coronal view; (C) Sagittal view illustrating the maximum thickness of the SM. (D) Axial view; (E) Coronal view; (F) Sagittal view depicting the diameter of the periapical translucency.

All measurements were independently performed by 2 calibrated examiners who were blinded to the clinical data, A subset of 25 scans was re-evaluated after a 2-week interval to assess intra- and inter-examiner reliability. The intraclass correlation coefficient (ICC) was excellent at 0.903, and the mean values from the 2 examiners were used for the final statistical analysis.

### Statistical analysis

Data distribution was assessed using the Shapiro–Wilk test. As SMT values were not normally distributed, nonparametric statistical methods were applied. Continuous variables are presented as mean ± standard deviation (SD) or median (interquartile range, IQR), based on distribution. The Mann–Whitney U test was used to compare SMT between 2 groups. The Kruskal–Wallis test was applied to evaluate differences in SMT among multiple CBCT-PAI categories. When appropriate, post hoc pairwise comparisons were performed with Bonferroni correction. Spearman’s rank correlation coefficient was calculated to assess the association between SMT and CBCT-PAI grades. All tests were 2-tailed, and a *P* value <.05 was considered statistically significant. All statistical analyses were conducted using IBM SPSS Statistics version 27.0 (IBM Corp.).

## Results

In accordance with the 2018 World Workshop classification, 330 patients (158 females and 172 males) diagnosed with Stage III to IV periodontitis were included (Stage III: 218; Stage IV: 112). Overall, patients with severe periodontitis exhibited increased SMT. Male patients had significantly greater maximal SMT values than females (*P* < .001). Although Stage IV patients showed slightly higher SMT values than Stage III patients, this difference was not statistically significant (*P* = .667).

When comparing the bilateral maxillary sinuses, no significant difference in maximal SMT was observed between Quadrant 1 and Quadrant 2 (*P* = .385), indicating a generally symmetrical mucosal thickening pattern in patients with severe periodontitis, as shown in Table 1.

**Table 1:** Associations between parameters and maximal SMT.

Parameters	N (%)	Maximal SMT (mm)	*P*-value
Sex (age)			
Female (51.7 ± 13.7)	158 (47.9)	4.63 (2.37, 8.45)	<.001*
Male (49.6 ± 12.8)	172 (52.1)	6.97 (3.58, 13.16)	
Stage (age)			
III (51.7 ± 13.0)	218 (66.1)	5.52 (2.68, 11.15)	.667
IV (48.5 ± 13.5)	112 (33.9)	6.09 (2,79, 11.76)	
Quadrant			
Quadrant 1	330 (100.0)	3.37 (1.68, 7.31)	.385
Quadrant 2	330 (100.0)	3.37 (1.69, 7.46)	

⁎ *P* < .001.

A total of 1816 maxillary posterior teeth were included in the tooth-level analysis. In both Stage III and Stage IV groups, statistically significant differences were observed among the 3 tooth positions for CBCT-PAI scores (Stage III: *P* < .001; Stage IV: *P* < .001) and SMT values (Stage III: *P* < .001; Stage IV: *P* < .001).

Post hoc pairwise comparisons demonstrated that the first molar (tooth 6) consistently presented the highest CBCT-PAI scores in both stages. Regarding SMT, both the first molar and second molar exhibited significantly greater mucosal thickness compared with the second premolar in Stage III and Stage IV (adjusted *P* < .05). Although SMT was present across all posterior teeth, its magnitude varied by tooth position, indicating a tooth-specific distribution pattern. At the same time, comparable patterns were observed between Quadrant 1 and Quadrant 2 within each stage, indicating bilateral mucosal thickening, as presented in Table 2.

**Table 2:** Comparison of CBCT-PAI scores and SMT among teeth.

Stage	Quadrant	Tooth position	N	CBCT-PAI†	Kruskal–Wallis test	SMT (mm)†	Kruskal–Wallis test
III	Quadrant 1	Second premolar	213	0 (0, 2)^a^	H = 34.09 *P <* .001*	1.52 (0.76, 3.94)^d^	H = 26.60 *P <* .001*
		First molar	205	2 (0, 3)^b^		2.24 (1.16, 5.37)^e^	
		Second molar	201	0 (0, 3)^a^		2.06 (1.20, 5.41)^e^	
	Quadrant 2	Second premolar	211	1 (0, 3)^a,b^		1.56 (0.68, 3.31)^d^	
		First molar	206	2 (0, 3)^a^		2.44 (1.11, 6.05)^e^	
		Second molar	201	2 (0, 3)^b^		2.13 (0.92, 4.51)^de^	
IV	Quadrant 1	Second premolar	100	1 (0, 2)^a^	H = 71.98 *P* < .001*	1.95 (0.92, 4.93)^d^	H = 21.85 *P* < .001*
		First molar	88	2 (1, 3)^b^		2.89 (1.24, 6.14)^d^	
		Second molar	94	2 (1, 3)^b^		2.24 (1.18, 6.93)^d^	
	Quadrant 2	Second premolar	105	1 (0, 2)^a^		1.36 (0.00, 5.74)^d^	
		First molar	95	2 (2, 3)^b^		2.88 (1.64, 7.12)^e^	
		Second molar	97	2 (1, 3)^b^		2.77 (1.32, 5.76)^e^	

For each measured parameter, pairwise comparisons were performed using the Mann-Whitney U test with Bonferroni correction; the significance level was set at P < 0.0167. Comparisons were conducted separately for left-side teeth and right-side teeth. Within each side, superscript lowercase letters indicate statistically significant differences between groups. For the CBCT-PAI score, letters an and b are used; for the SMT, letters d and e are used. Within a given side, the same letter denotes no statistically significant difference , whereas different letters denote a statistically significant difference. When a group is marked with two letters (e.g., “a, b”), this indicates that the group does not differ significantly from groups marked with “a” nor from those marked with “b”.

⁎ *P* < .001 indicates statistically significant differences between SMTs among different tooth positions within same stage.

† For each measured parameters, values with different superscript letters indicate significant differences between teeth within the same stage and same quadrant (Mann–Whitney U test with Bonferroni correction, *P* < .0167).

Tooth-level analysis of the association between CBCT-PAI and corresponding SMT demonstrated that SMT generally increased with higher CBCT-PAI scores, indicating a positive trend across the 1 to 5 grading range in both stage III and stage IV. The SMT corresponding to different CBCT-PAI score in stage IV was greater than that in stage III. The Kruskal–Wallis test revealed a statistically significant overall difference in SMT among the CBCT-PAI score (*P* < .001) in both stage III and stage IV. Furthermore, Spearman correlation analysis confirmed a modest but statistically significant positive association between CBCT-PAI and SMT (Stage III: ρ = 0.213, *P* < .001; Stage IV: ρ = 0.244, *P* < .001), supporting the relationship between increasing periapical severity and SMT, as presented in Table 3.

**Table 3:** Tooth-level analysis of the association between CBCT-PAI and corresponding SMT.

Stage	CBCT-PAI	N (%)	SMT (mm)	Spearman's rank	Kruskal–Wallis test
III	0	641 (51.8)	1.62 (0.74, 3.79)	ρ = 0.213 *P* < .001*	H = 62.30 *P* < .001*
	1	35 (2.8)	1.52 (0.90, 3.11)		
	2	181 (14.6)	1.93 (1.05, 4.34)		
	3	217 (17.5)	2.62 (1.39, 5.86)		
	4	133 (10.8)	3.41 (1.49, 8.87)		
	5	30 (2.4)	4.32 (1.70, 8.94)		
IV	0	114 (19.7)	1.75 (0.61, 6.70)	ρ = 0.244 *P* < .001*	H = 42.87 *P* < .001*
	1	175 (30.2)	1.64 (0.79, 4.00)		
	2	109 (18.8)	2.20 (1.21, 4.10)		
	3	94 (16.2)	3.00 (1.80, 7.32)		
	4	65 (11.2)	4.29 (1.88, 7.45)		
	5	22 (3.8)	6.25 (3.45, 9.41)		

⁎ *P* < .001.

## Discussion

This CBCT-based study evaluated SMT in patients diagnosed with Stage III-IV periodontitis complicated by apical periodontitis and identified two principal findings. First, SMT was prevalent and frequently bilateral in this cohort, suggesting the presence of diffuse inflammatory response in the posterior maxilla. This observation aligns with previous studies, indicating a trend toward increased SMT in cases of more severe periodontal destruction.^11^^,^^19^^,^^20^ Second, within this high-risk cohort, increasing severity of apical periodontitis was modestly but consistently associated with greater SMT, indicating a possible association within this generalised mucosal response. These findings may reflect the combined inflammatory burden present in advanced disease.

Chronic periodontal destruction in the posterior maxilla is characterised by persistent release of inflammatory mediator, vascular alterations and bone resorption. Given the thin bony partition and rich vascular communication between the posterior tooth roots and the sinus floor, inflammatory products may diffuse toward the Schneiderian membrane, which may be associated with reactive mucosal thickening. This diffuse inflammatory state may establish a baseline level of SMT, upon which localised lesions may impose additional localised changes.

Previous studies have established that the presence of apical periodontitis substantially elevates the risk of maxillary sinus mucosal thickening, with reported odds ratios several times higher than those observed in teeth without periapical lesions.^21^^,^^22^ In the present cohort, SMT in severe periodontitis complicated by apical periodontitis was more pronounced than values previously reported for severe alveolar bone loss alone, and SMT exhibited a distinct upward trend with increasing CBCT-PAI scores.^23^ Based on previous studies and the above finding, we hypothesise that apical periodontitis may exert an additional effect on SMT within our study population. Although the correlation coefficient was modest, this association remained consistent across both the stage III and stage IV groups. The severity of SMT has been reported to be positively associated with gender and the distance between the sinus floor and the root apex.^24^ Given the multifactorial nature of SMT and the fact that the study population was not limited to teeth with periapical lesions, our findings do not indicate a strong correlation.

Tooth-level analyses further revealed a site-specific distribution pattern. The maxillary first molar exhibited the highest CBCT-PAI scores and among the greatest SMT values. These findings are consistent with previous CBCT studies identifying the first molar as the most frequently affected tooth in odontogenic disease and odontogenic sinus alterations.^14^^,^^25^ Anatomically, the roots of the first molar are often in close proximity to the sinus floor, separated by only a thin cortical plate. Functionally, its early eruption and prolonged exposure to occlusal loading may be associated with cumulative periodontal attachment loss and potential secondary endodontic involvement.^26^^,^^27^ Studies have shown that the smaller the distance between the root apex and the sinus floor, the greater the risk of maxillary sinusitis.^22^ These combined anatomical and disease-related factors likely explain its prominent role in influencing sinus mucosal changes.^14^^,^^24^ Interestingly, although the maxillary first molar demonstrated the highest periapical severity, SMT values for the maxillary first molars and second molars were comparable within the same periodontal stage. In contrast, the second premolar exhibited significantly lower SMT values, likely due to its greater apical–sinus separation.^20^

Sex-related differences were also observed, with male patients showing significantly greater SMT than females, consistent with prior observations.^24^ As smokers were excluded from the analysis, this difference may reflect underlying biological variability or unmeasured environmental influences such as occupational irritants and ambient air quality.^28^ However, the present study was not designed to investigate these determinants, and further research is required to clarify their contribution.

From a clinical perspective, these findings suggest the potential value of comprehensive assessment of both periodontal and apical pathology in patients undergoing sinus augmentation. Preoperative CBCT assessment allows for identification of such combined pathology and may provide supportive information for risk stratification and surgical planning. Addressing active periodontal and endodontic infection prior to sinus elevation may be clinically relevant; however, given the retrospective nature of this study, it is not possible to draw conclusions regarding its impact on surgical outcomes.^6^^,^^8^

The use of CBCT combined with a standardised periapical grading system (CBCT-PAI) represents a methodological strength of this study.^29^ Three-dimensional imaging enables accurate assessment of lesion severity and sinus mucosal alterations that cannot be reliably detected with 2-dimensional radiography.^30^^,^^31^ The use of maximum SMT values reduces the risk of underestimating focal inflammatory changes and has been applied in CBCT-based assessments. In CBCT-based studies, the grading criteria for SMT proposed by Di Girolamo et al are based entirely on the maximum thickness to guide clinical decision-making.^16^ Therefore, capturing the maximum thickness provides a pragmatic indicator of potential surgical risk. The relatively large sample size and the application of the 2018 World Workshop classification further enhance the internal consistency of the study population.

Nevertheless, several limitations should be acknowledged. The number of Stage IV cases and high CBCT-PAI scores were relatively small. Future investigations will aim to expand the sample size and incorporate cohorts with isolated periodontitis or apical periodontitis as controls, employing multivariate modelling to better elucidate the underlying mechanisms. Additionally, the present study did not quantify specific anatomical variables – such as the distance between the root apex and the sinus floor – that may influence SMT.^20^ The CBCT-PAI utilised relies predominantly on lesion diameter, which may not adequately capture volumetric dimensions or morphological complexity.^20^^,^^24^^,^^29^ These limitations could potentially restrict the ability to accurately reflect the true pathological burden.^32^ Therefore, our ongoing research will incorporate the distance between the root apex and the sinus floor and lesion volume to further investigate their influence on SMT. Finally, the retrospective design inherently constrains our ability to establish temporal relationships or assess mucosal alterations following periodontal or endodontic treatment. We plan to conduct prospective studies in the future to evaluate the longitudinal impact of periodontal and endodontic interventions on the maxillary sinus, thereby providing a more robust evidence base for clinical management.

In summary, in patients with Stage III-IV periodontitis, SMT appears to be associated with both diffuse and localised inflammatory patterns observed within this cohort. The maxillary first molar represents a key site where odontogenic inflammation interacts with the sinus. These findings highlight the potential role of CBCT-based assessment and emphasise the importance of integrated periodontal and endodontic management in treatment planning for posterior maxillary rehabilitation.

## Conflict of interest

The authors declare that they have no known competing financial interests or personal relationships that could have appeared to influence the work reported in this paper.
